# Impact of Pre-Transplant Anti-T Cell Globulin (ATG) on Immune Recovery after Myeloablative Allogeneic Peripheral Blood Stem Cell Transplantation

**DOI:** 10.1371/journal.pone.0130026

**Published:** 2015-06-22

**Authors:** Sophie Servais, Catherine Menten-Dedoyart, Yves Beguin, Laurence Seidel, André Gothot, Coline Daulne, Evelyne Willems, Loïc Delens, Stéphanie Humblet-Baron, Muriel Hannon, Frédéric Baron

**Affiliations:** 1 Hematology Research Unit, GIGA-I3, University of Liège, Liège, Belgium; 2 Bone Marrow Transplantation Unit, Department of Clinical Hematology, CHU of Liège, Liège, Belgium; 3 Department of biostatistics, SIMÉ, CHU of Liège, Liège, Belgium; 4 Department of Laboratory Medicine, CHU of Liège, Liège, Belgium; Beth Israel Deaconess Medical Center, Harvard Medical School, UNITED STATES

## Abstract

**Background:**

Pre-transplant infusion of rabbit anti-T cell globulin (ATG) is increasingly used as prevention of graft-versus-host disease (GVHD) after allogeneic peripheral blood stem cell transplantation (PBSCT). However, the precise impact of pre-transplant ATG on immune recovery after PBSCT is still poorly documented.

**Methods:**

In the current study, we compared immune recovery after myeloablative PBSCT in 65 patients who either received (n = 37) or did not (n = 28) pre-transplant ATG-Fresenius (ATG-F). Detailed phenotypes of circulating T, B, natural killer (NK) and invariant NKT (iNKT) cells were analyzed by multicolor flow cytometry at serial time-points from day 40 to day 365 after transplantation. Thymic function was also assessed by sjTREC quantification. Serious infectious events were collected up to 2 years post-transplantation.

**Results:**

Pre-transplant ATG-F had a prolonged (for at least up to 1-year) and selective negative impact on the T-cell pool, while it did not impair the recovery of B, NK nor iNKT cells. Among T cells, ATG-F selectively compromised the recovery of naïve CD4^+^, central memory CD4^+^ and naïve CD8^+^ cells, while it spared effector memory T and regulatory T cells. Levels of sjTRECs were similar in both cohorts at 1-year after PBSCT, suggesting that ATG-F unlikely impaired thymopoiesis at long-term after PBSCT. Finally, the incidence and rate of serious infections were similar in both groups, while ATG-F patients had a lower incidence of grade II-IV acute graft-versus-host disease.

**Conclusions:**

Pre-transplant ATG-F induces long-lasting modulation of the circulating T-cell pool after myeloablative PBSCT, that may participate in preventing graft-versus-host disease without deeply compromising anti-pathogen defenses.

## Introduction

The use of peripheral blood stem cells (PBSC) instead of bone marrow as graft source for allogeneic stem cell transplantation has resulted in increased incidences of both grade III-IV acute and extensive chronic graft-versus-host disease (GVHD) [1]. This prompted several groups of investigators to assess the ability of pre-transplant infusion of rabbit anti-T cell globulins (ATG) to prevent GVHD after PBSC transplantation (PBSCT) [2–7]. Rabbit ATG are polyclonal antibody preparations corresponding to the purified IgG fraction of sera from rabbits that were immunized with human T cells. Due to their relatively long half-life in human plasma (up to 6 weeks), ATG preparations can persist in blood for several weeks after infusion [8, 9] and destroy donor T cells passively transferred with the graft.

Effects of pre-transplant ATG on GVHD prevention after stem cell transplantation have been demonstrated in a number of recent studies [2–7]. Most of the studies performed in patients given myeloablative conditioning have shown that ATG decreased the incidence of both acute and chronic GVHD, without increasing relapse risk [3, 5, 6]. Similarly, ATG has been reported to successfully prevent GVHD after reduced intensity conditioning (RIC) transplantation, while its impact on relapse incidence in that setting remained controversial. Hence, in a large CIBMTR study including patients who underwent RIC transplantation for various hematological malignancies, Soiffer et al. reported that ATG was associated with a higher risk of relapse [7]. To the contrary, in a large EBMT study of patients given RIC PBSCT for acute myeloid leukemia, the authors observed that ATG did not result in a higher relapse risk, unless if it was given at high doses [2]. Eventually, in both the RIC and myeloablative settings, concerns have also risen about a higher incidence of infectious complications with ATG, specifically when used at high doses [10–12].

The most widely used rabbit ATG preparations in Europe are ATG-T (Thymoglobulin, Genzyme/Sanofi) that is produced by rabbit immunization against human thymocytes, and ATG-F (Fresenius/Neovii) that is produced by rabbit immunization against the human T lymphoblastoid cell line Jurkat [13]. Both ATG preparations contain a diverse spectrum of antibody specificities directed against T-cell epitopes that can mediate T-cell depletion in blood and in lymphoid tissues through induction of activation-associated apoptosis and complement- or natural killer (NK)-cell-dependent lysis. However, because of their polyclonal nature, ATG preparations do not exclusively target T cells, but also other immune cells. Hence, ATG-T contains antibodies against multiple antigens that are expressed on various subsets of T, B, NK, granulocyte, monocyte/macrophage and dendritic cells, as well as on thymic stromal cells [13, 14]. The precise spectrum of ATG-F has not been studied extensively yet. However, since ATG-F is produced by immunization against homogeneous Jurkat cells, it is likely that its spectrum is smaller (e.g. anti-CD4 and anti-HLA-DR antibodies are lacking in ATG-F [13]). This may be relevant given recent evidences of an immunoregulatory activity of several anti-CD4 antibodies [15]. Further, in contrast to ATG-T [14], ATG-F does not include antibodies directed against thymic stromal cells. Therefore, the impact of ATG on thymic function may likely differ according to ATG formulation.

Despite being increasingly used, the impact of ATG on immune recovery after PBSCT has not been widely studied. Further, most published studies investigated ATG-T [16–18]. In the largest published study, Bosch *et al*. observed that ATG-T delayed the recovery of naive and memory CD4^+^T cells as well as of naive CD8^+^T cells after myeloablative PBSCT.[16] Other groups of investigators have observed a negative impact of ATG-T on naive T-cell output by the thymus after PBSCT [17, 19]. Only few studies have specifically analyzed the effects of ATG-F on immune recovery after stem cell transplantation [20, 21]. In a pediatric population, Mensen et al. retrospectively showed that ATG-F patients had a similar long-lasting T-cell deficiency as patients given ATG-T at standard doses [20]. However, they did not analyze T-cell subpopulations in details. Recently, Roll et al. focused on the effects of ATG-F on B cell recovery after allogeneic transplantation, and suggested that ATG-F also negatively impacted early memory B-cell reconstitution [21]. Here, we report one of the largest studies assessing the impact of ATG-F on the recovery of a large variety of lymphoid cell subsets as well as on thymic function after myeloablative PBSCT.

## Patients and Methods

### Patients

We performed a retrospective study of patients who underwent a first allogeneic PBSCT without *ex-vivo* T-cell depletion after myeloablative conditioning at our institution between January 2000 and September 2012. Myeloablative conditioning regimens were defined as those based on high dose total body irradiation (TBI) (≥ 500 cGy single dose or ≥ 800 cGy fractionated dose), high doses of Busulfan (> 8 mg/kg orally) or high doses of other alkylating agents [22]. Grafts were obtained from HLA-identical sibling donors or 9-10/10 HLA-A,-B,-C,-DR,-DQ unrelated donors. According to institutional guidelines, pre-transplant ATG-F was not given before year 2003. Between 2003 and 2006, it was only used in patients transplanted for non-malignant diseases. Since 2006 and thereafter, use of pre-transplant ATG-F was given to all patients with the exception of those with very high-risk malignant diseases. ATG-F was generally given for 3 consecutive i.v. injections (15mg/kg on days 3, 2 and 1 before transplantation). All patients provided written informed consent for use of protected health data for research and for blood sample collection, in accordance with the Declaration of Helsinki. Written informed consent from caretakers was obtained on behalf of the minor patients. This study was approved by our institutional review board (Comité d’Ethique Hospitalo-Facultaire Universitaire de Liège, study number: B70720096240).

Immune analyses were also performed on blood samples from 22 healthy age-matched volunteers, after having obtained their written informed consent.

### Clinical monitoring after PBSCT

T-cell chimerism was assessed at days 40, 100, 180, 365 after PBSCT and yearly thereafter. Chimerism analyses were carried out after T-cell isolation with the RosetteSep technology (StemCell Technologies, Vancouver, Canada) and were performed using either fluorescence in situ hybridization of sex chromosomes in cases of sex-mismatched donor/recipient pairs or PCR-based analysis of polymorphic microsatellite regions (multiplex PCR) in cases of sex-matched donor/recipient pairs. Graft rejection was defined as occurrence of < 5% donor T-cell chimerism. Disease evaluation was also routinely carried out on days 40, 100, 180, 365 after PBSCT and yearly thereafter.

Postgrafting immunosuppression mostly consisted of a calcineurin inhibitor (cyclosporine or tacrolimus) combined with methotrexate. Acute GVHD was graded using the 1994 consensus conference standard criteria [23]. Chronic GVHD was diagnosed based on diagnostic or distinctive features and was graded in mild, moderate or severe form, as proposed by the 2005 National Institutes of Health (NIH) Consensus criteria [24]. Grade 2–4 acute GVHD as well as moderate/severe chronic GVHD were usually treated with prednisolone as first line therapy, supplemented by other immunosuppressive drugs for some patients.

All patients were treated in laminar airflow or high-efficiency particulate absorption air filtered rooms during the early post-transplant period, until they had an absolute neutrophil count above 1 x 109/L. Infection prophylaxis usually consisted of levofloxacine, fluconazole, acyclovir and co-trimoxazole or aerosolized pentamidine (**S1 Methods**). Patients were screened weekly for CMV by antigenemia and/or PCR in blood. Pre-emptive therapy with ganciclovir was initiated after a positive antigenemia or PCR and discontinued after at least two consecutive negative results. Epstein-Barr virus (EBV) viral load was not assessed before 2006. Since 2006, it was quantified by PCR upon clinical suspicion and therapy with rituximab was initiated if it was > 10,000 copies/mL. A pet-CT was usually performed in these cases, in order to exclude EBV-mediated post-transplant lymphoproliferative disorder (PTLD).

### Serious infections after PBSCT

Only serious infections, potentially associated with clinical compromise, were considered in this analysis. They were defined, as previously described [25]:

- **Serious bacterial infections-**These included bacterial infections of any organ site requiring i.v. therapy and/or hospitalization. Bacterial infections were either microbiologically proven or presumed based on the combination of clinical presentation and response to treatment with antibiotics. Bacteremia by coagulase-negative staphylococci, Micrococcus spp and saprophytic Corynebacterium spp were excluded in this analysis, as well as infections of intravascular devices because of possible exogenous contamination and potential for reporting bias.
- **Serious viral infections**–These included CMV and EBV viremia requiring treatment, and other documented invasive viral infections requiring i.v. therapy and/or hospitalization (such as disseminated form or visceral involvement of herpes family virus infections, adenovirus disease, B or C viral hepatitis and lower respiratory tract infections by respiratory viruses). CMV disease was diagnosed according to previously reported criteria [26].
- **Serious fungal infections**–These included any invasive fungal infections. Invasive aspergillosis was defined as proven, probable or possible according to standard criteria [27]. Interstitial pneumonia caused by Pneumocystis Jiroveci was considered as fungal infection.
- **Serious parasitic infections**–These included toxoplasmosis and other invasive parasitic infections. Infection-related mortality was defined as death from infection as the primary cause of death, basing on the Copelan hierarchical scheme for causes of death assignment [28].

### Immune recovery monitoring

#### Phenotypes of circulating lymphocytes by flow cytometry

Basic phenotypes of circulating lymphocytes was prospectively assessed by flow cytometry on fresh heparinized peripheral blood samples collected on days 40, 60, 80, 100, 120, 180 and 365 after PBSCT. Cells were characterized using 4-color flow cytometry after red blood cell lysis, as previously described [29, 30].

More detailed lymphoid cell phenotypes were retrospectively assessed on cryopreserved peripheral blood mononuclear cells (PBMCs) that were prospectively collected on days 40, 100, 180 and 365 after PBSCT, as previously described [31]. Briefly, PBMCs were isolated by density gradient centrifugation (Ficoll-Paque; GE Healthcare) from fresh heparinized blood samples and then frozen. Lymphocyte subsets were analyzed using 8-color flow cytometry. The following antibodies were used: anti-CD4 PercP (SK3), anti-CD45RA APC (HI 100), anti-CD31 PE (L133.1), anti-CD19 APCCy7 (SJ25C1), anti-IgD biot (IA6-2), anti-CD27 PE (M-T271), Anti-CD3 V500 (SP34-2), anti-CD56 V450 (B159) and anti-CD16 FITC (3G8) from BD Biosciences (San Diego, CA, USA); anti-CD8 FITC (HiT8a), anti-CCR7 PeCy7 (3D12), anti-CD25 PE (BC96), anti-CD127 biot (eBioRDR5), anti-TCRVα24Jα18 PE (6B11), streptavidine eF450, streptavidine APCCy7 and streptavidine PeCy7 from e-Bioscience (San Jose, CA, USA); anti-FoxP3 FITC 206D from Biolegend (San Diego, CA, USA); anti-TCR Vβ11 biot (C21) from Analis (Namur, Belgium). Intracellular staining was performed using human intracellular FoxP3 staining kit (Biolegend, San Diego, CA, USA) according to the manufacturer’s instructions. Lymphoid cells were gated on forward versus side-scatter plots. Cells were acquired on a FACSCanto II (Becton Dickinson) and data were analyzed with FlowJo software (7.0, Tree Star Inc., San Carlos, CA). A minimum of 100 events in the parent population was considered mandatory to ensure reliable subset analyses and data were not considered if the number of analyzed cells was not sufficient. Cell counts at each time-point after PBSCT were calculated as absolute lymphocyte counts measured in peripheral blood multiplied by cell percentages obtained in the lymphoid gate.

#### T-cell receptor excision circles (sjTRECs) assay

SjTREC quantification was retrospectively performed on cryopreserved PBMCs that were prospectively collected on days 100 and 365 after PBSCT. SjTRECs were quantified by nested real-time PCR, as previously described (**S2 Methods**) [29, 30, 32].

### Statistical analyses

Clinical outcomes were overall survival (OS), non-relapse mortality (NRM), disease relapse, clinically significant acute (grade II-IV) and moderate/ severe chronic (NIH 2–3) GVHD, and infections (bacterial, viral, fungal infections and infection-related mortality). NRM was considered as time to any death occurring before disease relapse. Clinical outcomes were estimated using Kaplan-Meier product-limit estimator for overall survival and cumulative incidence functions were estimated for competing risks analyses. NRM and relapse were considered to be mutually competing risks. Death was considered as a competing risk for acute and chronic GVHD. Death and relapse were considered as competing risks for infection. Comparisons of survival and cumulative incidence functions were performed by log-rank test.

Data concerning immune recovery monitoring were censored at time of relapse/progression of the underlying disease. The Mann-Whitney rank sum test was used to compare immune data between ATG-F and control patients, with a *p*-value <0.05 (2-tailed) considered as significant. Stepwise multiple regression analyses were performed to identify potential factors affecting cell subset counts at days 40, 100, 180 and 365. Logarithmic transformation was performed for cell counts before these analyses, to normalize their distribution. Because of high disequilibrium in ATG-F and control patient distribution according to date of transplantation, we could not enter the period of PBSCT as a covariate in our analysis. To minimize chance of spurious associations because of multiple comparisons, *p* <0.01 was considered significant. If pretransplant factors were significantly associated with the posttransplant immune cell counts, we assumed a cause and effect relationship.

Statistical analyses were performed using GraphPad Prism (GraphPad Software, San Diego, CA) and SAS version 9.3 (SAS Institute, Cary, NC, USA).

## Results

### Patient characteristics and clinical outcomes

A total of 65 consecutive patients met inclusion criteria: 37 received pre-transplant ATG-F and 28 did not (control group). Main patient and transplantation characteristics are summarized in **Table 1**. ATG-F was administered at a total dose of 45mg/kg in all but 3 ATG-F patients. The two (control and ATG-F) cohorts were balanced for recipient age, donor age, donor type and donor/recipient HLA-matching, donor/recipient CMV serostatus, graft composition, conditioning regimen and postgrafting immunosuppression. In accordance with our policy concerning use of ATG-F, there was higher proportions of patients with high or very high risk disease (as defined by the Disease Risk Index, DRI[33]) in the control group. As also expected, because of changes in clinical practice concerning use of pre-transplant ATG-F during the study period, most patients transplanted before 2006 did not receive ATG-F whereas the majority of patients transplanted in 2006–2012 received ATG-F. This resulted in an unbalanced repartition of patients between the two cohorts for the period of transplantation. Median follow-up from PBSCT in surviving patents was 1418 days (range, 843 to 2497) for the global cohort.

**Table 1:** Patients characteristics.

	Control (N = 28)	ATG-F (N = 37)	*p*-value
**Patient age, median (range), years**	35 (4–55)	40 (4–53)	0.24
**Patient gender, male, # of patients (%)**	20 (71)	23 (62)	0.43
**Disease at transplantation, # of patients**			*
Acute myeloid leukemia	11	18	
Acute lymphoblastic leukemia	7	9	
Myeloproliferative disorder	2	1	
Myelodysplatic syndrome	2	3	
Non-Hodgkin’s lymphoma	4	2	
Hodgkin’s lymphoma	1	1	
Multiple myeloma	1	1	
Aplastic anemia	0	2	
**Disease risk at transplantation** ^**a**^ **, # of patients (%)**			0.012
Non malignant disease	0 (0)	2 (5)	
DRI, low	1 (4)	4 (11)	
DRI, intermediate	7 (25)	16 (43)	
DRI, high	14 (50)	15 (41)	
DRI, very high	6 (21)	0 (0)	
**Donor age, median (range), years**	28 (4–58)	32 (4–54)	0.66
**Donor gender, male, # of patients (%)**	18 (64)	20 (54)	0.41
**Donor type, # of patients (%)**			
Sibling (HLA-identical)	11 (39)	18 (49)	0.45
Unrelated	17 (61)	19 (51)	
**Donor-recipient HLA status, # of patients (%)**			
HLA-matched	21 (75)	29 (78)	0.75
HLA-mismatched	7 (25)	8 (22)	
**CMV serostatus, donor/patient, # of patients (%)**			
-/-	11 (39)	13 (35)	0.73
Others	17 (61)	24 (65)	
**Graft composition, x 10** ^**6**^ **/kg recipient**			
CD34; median (range)	4.87 (1.32–32.24)	5.25 (0.38–30.74)	0.32
Nucleated cells; median (range)	15.67 (3.35–222.64)	15.88 (4.86–59.12)	0.59
**Conditioning regimen, # of patients (%)**			0.15
TBI-based (800–1200 cGy)	27 (96)	30 (81)	
Busulfan-based (16mg/kg, orally)	1 (4)	4 (11)	
Others	0 (0)	3 (8)	
**Pre-transplant ATG-F, # of patients (%)**			NA
15mg/kg days -3, -2, -1	NA	34 (92)	
20mg/kg day -1	NA	1 (3)	
30mg/kg days -5, -4, -3	NA	2 (5)	
**Postgrafting immunosuppression, # of patients (%)**			
Cyclosporine + Methotrexate	21 (75)	25 (68)	0.24
Tacrolimus + Methotrexate	2 (7)	8 (22)	
Cyclosporine or tacrolimus alone	5 (18)	4 (11)	
**Year of transplantation, # of patients (%)**			<0.0001
2000–2005	17 (61)	1 (3)	
2006–2012	11 (39)	36 (97)	
**Neutrophil engraftment** ^**b**^ **, median (range), days**	14.5 (10–19)	17 (11–26)	0.0016
**100-day CIfof grade II-IV acute GVHD; % (95% CI)**	64.3 (43.0–79.4)	16.2 (6.5–29.9)	<0.0001
**2-yr CIf of moderate/severe chronic GVHD; % (95% CI)**	32.1 (15.4–50.2)	27.3 (14.0–42.5)	0.1265
**2-yr CIf of relapse; % (95% CI)**	32.1 (15.7–49.9)	21.6 (10.0–36.1)	0.0987
**2-yr CIf of non relapse mortality; % (95% CI)**	42.9 (24–60.5)	13.5 (4.8–26.6)	0.0023
**2-yr overall survival; % (95% CI)**	32.1 (16.1–49.3)	72.9 (55.4–84.4)	0.0011
**Median follow-up, days, median (range)**	1502 (936.5–2497)	1372 (843–2098)	0.58

CIf indicates cumulative incidence function; NA, not applicable.

* No statistical test is provided due to small sample size.

^a^ classified according to Armand et al. [33].

^b^ Absolute neutrophil counts >0.5 x10^9^/L for at least consecutive 3 days.

No patient experienced primary engraftment failure. Time for neutrophil recovery was rapid in both groups, although slightly delayed in the ATG-F group. Rapid donor T-cell engraftment was observed in both groups, with median donor T-cell chimerism levels being above 95% on day 40 after PBSCT in both groups (**S1 Fig**). Compared to control patients, ATG-F patients experienced better overall survival, lower incidence of non-relapse mortality and similar incidence of relapse at 2 years after PBSCT (**Table 1 and S1 Fig**). These results should however be interpreted with caution, given that the choice of giving or not ATG-F was not random. Confounding factors in our analysis include transplant period and disease risk. They also had a dramatically lower incidence of acute GVHD at 100 days while the 2-year incidence of chronic GVHD was similar in both groups.

### Circulating lymphoid cells–Basic phenotypes (4-color flow cytometry)

**Fig 1:**
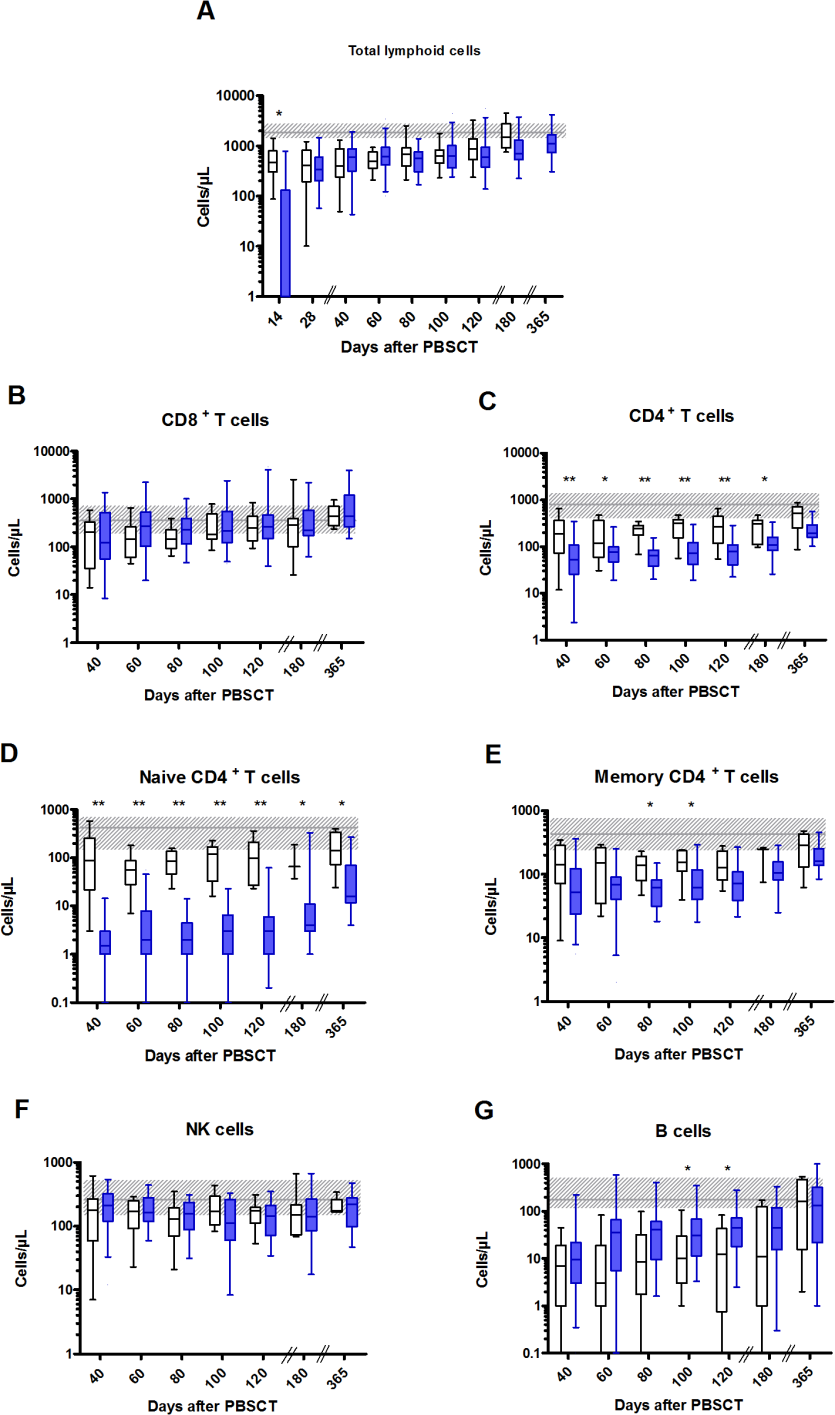
Lymphoid cell recovery after PBSCT with or without pre-transplant ATG-F. Levels of circulating lymphoid cells in the peripheral blood of ATG-F (blue box) and control (white box) patients are shown. Cell phenotypes were assessed for 27/34, 27/33, 27/31, 26/30, 24/30, 26/29 and 19/25 disease-free survivors in the ATG-F cohort on days 40, 60, 80, 100, 120, 180 and 365 after PBSCT, respectively; and for 17/23, 11/19, 10/17, 12/17, 11/17, 8/15 and 5/9 disease-free survivors in the control cohort on days 40, 60, 80, 100, 120, 180 and 365 after PBSCT, respectively. Box and whisker plots display the median, 25^th^ and 75^th^percentiles of the distribution (box) and whiskers extend to 5^th^ and 95^th^ percentiles. The grey horizontal line and shaded grey area show the median and normal range (from 5^th^ to 95^th^ percentile) in 22 age-matched healthy controls. *p <0.05; ** *p*<0.01.

Recovery of lymphoid cell subsets in the peripheral blood is shown in **Fig 1A–1F**. ATG-F and control patients had similar levels of circulating lymphoid cells during the first year after PBSCT, except on day 14 when they were lower in ATG-F patients. By assessing lymphoid cell subsets, we did not observe any difference in levels of NK cells (CD3^-^CD56^+^) and CD8^+^T cells (CD3^+^CD8^+^) between ATG-F and control patients over the first year after PBSCT, with rapid recovery of these cells in both groups (medians reaching normal values by 40–100 days after PBSCT). To the contrary, CD4^+^T cells (CD3^+^CD4^+^) recovered more slowly in both groups. Further, a more profound CD4^+^T-cell depletion was observed in ATG-F patients the first 6 months after PBSCT, mostly due to slower recovery of naive (CD3^+^CD4^+^CD45RA^+^) cells. Counts of memory CD4^+^T cells (CD3^+^CD4^+^CD45RO^+^) were roughly similar in both groups. Regarding B-cell (CD19^+^) recovery, median B-cell counts reached normal values 1 year after PBSCT in both groups. There was a trend for higher B-cell levels in ATG-F patients, although the difference reached statistical significance only on days 100 and 120 after PBSCT.

### Circulating lymphoid cells–Detailed phenotypes (8-color flow cytometry)

#### Naive and memory CD4^+^ and CD8^+^ T cells

**Fig 2:**
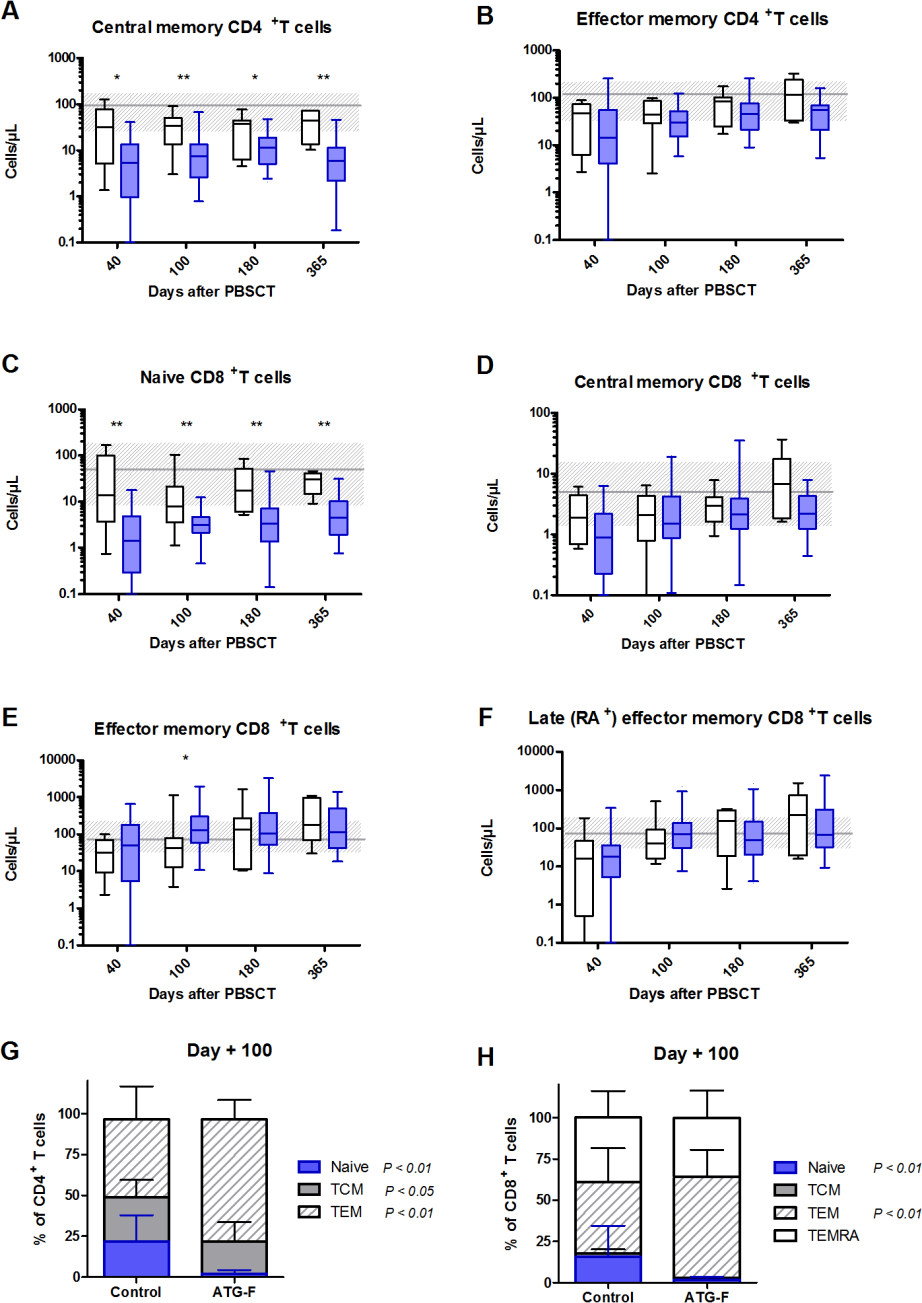
Recovery of CD4^+^ and CD8^+^ T-cell subsets after PBSCT with or without pre-transplant ATG-F. Circulating immune cells phenotypes were assessed for 31/34, 26/30, 28/29 and 20/25 disease-free survivors in the ATG-F cohort on days 40, 100, 180 and 365 after PBSCT, respectively; and for 6/23, 15/17, 9/15 and 7/9 disease-free survivors in the control cohort on days 40, 100, 180 and 365 after PBSCT, respectively. A-F) Absolute cell counts in the peripheral blood of ATG-F (blue box) and control (white box) patients are shown. Box and whisker plots display the median, 25^th^ and 75^th^ percentiles of the distribution (box) and whiskers extend to 5^th^ and 95^th^ percentiles. The grey horizontal line and shaded grey area show the median and normal range (from 5^th^ to 95^th^ percentile) in 22 age-matched healthy controls. G-H) Relative proportions of naïve and memory subsets among CD4+ (G) and CD8+ (H) T compartments were assessed, according to pre-transplant ATG-F or not. Mean and standard deviation are shown. *p <0.05; ** *p*<0.01.

We further evaluated CD4^+^ and CD8^+^T-cell subsets by analyzing CD45RA and CCR7 expression (**Fig 2A–2F**). Similar results were observed for absolute counts of naive (CD45RA^+^CCR7^+^) CD4^+^T cells as those obtained by using 4-color flow cytometry. ATG-F patients also had delayed recovery of circulating central memory (CD45RA^-^CCR7^+^) CD4^+^T cells up to 1 year after PBSCT. Moreover, although median levels were within normal values by day 40 in control patients, they did not reach the normal range in ATG-F patients by 1 year after PBSCT. To the contrary, effector memory (CD45RA-CCR7-) CD4^+^T cells reconstituted rapidly in ATG-F and control patients (medians within normal values on day 40–100 after PBSCT) and no difference was observed between the two groups.

When looking at the relative proportion of naive and memory subsets among CD4^+^T cells, we observed a specific defect of the naive and central memory cell compartments during the first 6–12 months after PBSCT in ATG-F patients as compared to control patients (*p*<0.01 on days 40, 100, 180 and <0.05 on day 365 for the naive subset, and *p*<0.05 on days 100 and 180 for the central memory subset). This resulted in compensatory higher proportion of effector memory cells (*p*<0.01 on days 40, 100, 180 and <0.05 on day 365). A representative diagram at day 100 is shown in **Fig 2G**.

Regarding CD8^+^T cells, median levels of central memory (CD45RA^-^CCR7^+^), effector memory (CD45RA^-^CCR7^-^) and CD45RA-re-expressing late effector memory (CD45RA^+^CCR7^-^) CD8^+^T cells were similar in both groups throughout the study period. Their median counts reached normal values by day 40–100 after PBSCT. To the contrary, a significant delay in recovery of naive (CD45RA^+^CCR7^+^) CD8^+^ T cells was observed up to 1 year after PBSCT in ATG-F patients as compared to control patients. Specifically, while median naive CD8^+^T-cell counts were within the normal range by day 40 in control patients, it did not reach the lower limit of normal range by 1 year after PBSCT in ATG-F patients.

Similarly to what was observed among CD4^+^T cells, we observed a reduced naive cell compartment and larger effector memory cell compartment among CD8^+^T cells in ATG-F patients during the first 6 months after PBSCT (*p*<0.01 on days 40, 100 and 180 for naive subset, and *p*<0.01 on day 100 and <0.05 at day 40 and 180 for effector memory subset). No difference was observed for central memory and CD45RA-re-expressing late effector memory compartments among CD8^+^T cells. A representative diagram at day 100 is shown in **Fig 2H**.

#### Regulatory T cells (Treg)

Treg (CD4^+^CD127^neg/low^FoxP3^+^) were also analyzed. No difference was observed in either their absolute number or their relative proportion among CD4^+^T cells between ATG-F and control patients (**S2A and S2B Fig**). Their recovery after PBSCT was slow in both groups, with the median of their absolute levels reaching normal values only by day 365 in control patients and lagging below the lower limit of normal range for at least 1 year in ATG-F patients (*p* = NS).

#### B cells, NK cells and invariant NKT cells (iNKT)

Recovery of total B cells (CD19^+^) and their naive (CD27^-^IgD^+^), unswitched memory [CD27^+^IgD^+^] and switched memory [CD27^+^IgD^-^] subsets was mainly superimposable in ATG-F and control patients (**S2C–S2E Fig**). Contrary to what was observed for T-cell subsets, most of circulating B cells were of naive phenotype during the first year after PBSCT. Naive B-cell counts recovered at day 365 whereas switched memory B-cell recovery was not achieved by 1 year after PBSCT.

NK-cell recovery was similar in both groups, with faster recovery of CD56^bright^NK cells (CD3^-^CD56^bright^CD16^-^) in comparison with CD56^dim^NK cells (CD3^-^CD56^dim^ CD16^+^) (**S2F and S2G Fig**).

Finally, as observed in healthy donors, levels of iNKT cells (CD3^+^CD56^+^TCR-Vα24Jα18^+^TCR-Vβ11^+^) were very low in the peripheral blood of transplanted patients. Medians were within normal values throughout the study period, except at day 40 for ATG-F patients when it lagged below the lower limit of normal range (*p*<0.05 compared to control patients) (**S2H Fig**).

### Circulating recent thymic emigrants (RTE) (sjTREC level quantification and flow cytometry)

**Fig 3:**
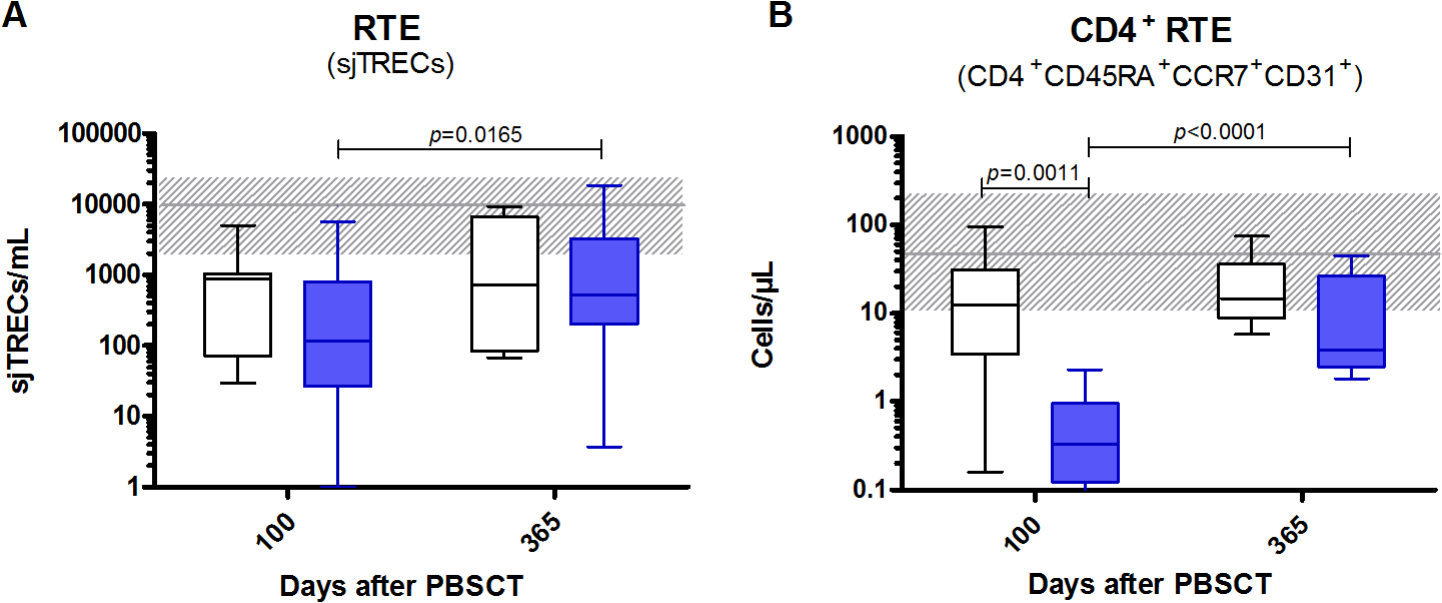
Circulating recent thymic emigrants after PBSCT with or without pre-transplant ATG-F. Levels of circulating RTE in the peripheral blood of ATG-F (blue box) and control (white box) patients are shown. A) RTE were assessed by sjTRECs quantification assay for 30/30 and 23/25 disease-free survivors in the ATG-F cohort on days 100 and 365 after PBSCT, respectively; and for 11/17 and 7/9 disease-free survivors in the control cohort on days 100 and 365 after PBSCT, respectively. B) RTE were assessed by flow cytometry for 26/30 and 20/25 disease-free survivors in the ATG-F cohort on days 100 and 365 after PBSCT, respectively; and for 15/17 and 7/9 disease-free survivors in the control cohort on days 100 and 365 after PBSCT, respectively. Box and whisker plots display the median, 25^th^ and 75^th^ percentiles of the distribution (box) and whiskers extend to 5^th^ and 95^th^ percentiles. The grey horizontal line and shaded grey area show the median and normal range (from 5^th^ to 95^th^ percentile) in 22 age-matched healthy controls.

Since naive T-cell subset recovery appeared to be severely affected in ATG-F patients, we further delineated the presence of circulating RTE, in order to determine the thymic output of naive T cells. Levels of RTE were analyzed both by sjTREC quantification for total T cells and by flow cytometry for CD4^+^RTE (CD4^+^CD45RA^+^CCR7^+^CD31^+^), with a significant correlation between the two techniques (p = 0.005) (**Fig 3**). There was a trend for lower sjTREC counts on day 100 in ATG-F compared to control patients (*p* = 0.09), but the difference receded on day 365 and sjTRECs levels significantly increased from day 100 to day 365 in ATG-F patients. Similar results were observed for CD4^+^RTE by flow cytometry, but the difference was significant on day 100 between ATG-F and control patients using this method assessing only the CD4^+^T-cell compartment. Overall, these results suggest that ATG-F unlikely impaired thymopoiesis from day 100 to 1 year after PBSCT.

### Multiple regression analysis of factors affecting CD4^+^ and CD8^+^ T-cell subset recovery

In order to evaluate whether pre-transplant ATG-F indeed independently impacted levels of CD4^+^T cells (total, naive and central memory cells) and naive CD8^+^T cells over the first year after PBSCT, we performed multiple regression analyses. Tested co-variables included pre-transplant parameters and occurrence of grade II-IV acute GVHD (before the day of immune cell assessment) because of the significant difference we observed in its cumulative incidence between ATG-F and control patients (see above). Results are presented in **Table 2**. Interestingly, ATG-F was the sole factor significantly impacting both short- and long-term (from day 40–100 to day 365) recovery of naive CD4^+^ and CD8^+^T cells and of central memory CD4^+^T cells. It also significantly impacted short-term (days 40 and 100) total CD4^+^T-cell levels. Similarly, acute GVHD had a negative impact on total CD4^+^ T-cell counts.

**Table 2:** Multivariate analysis of factors impacting immune cell levels after myeloablative PBSCT.

Immune cell subsets		Significant factors	Coefficient	(SE)	*p*
**Total CD4** ^**+**^ **T cells**	**day 40**	ATG-F	-1.55	(0.36)	<0.0001
		Acute GVHD	-1.22	(0.37)	0.0024
	**day 100**	ATG-F	-1.22	(0.24)	<0.0001
	**day 180**	-			
	**day 365**	-			
**Naive CD4** ^**+**^ **T cells**	**day 40**	ATG-F	-3.42	(0.40)	<0.0001
		Recipient’s age	-0.036	(0.012)	0.0070
	**day 100**	ATG-F	-3.42	(0.47)	<0.0001
	**day 180**	ATG-F	-2.52	(0.73)	0.0020
	**day 365**	ATG-F	-1.74	(0.60)	0.0089
**Central memory CD4** ^**+**^ **T cells**	**day 40**	-			
	**day 100**	ATG-F	-1.44	(0.35)	0.0003
	**day 180**	-			
	**day 365**	ATG-F	-1.81	(0.60)	0.006
**Naive CD8** ^**+**^ **T cells**	**day 40**	ATG-F	-2.47	(0.82)	0.0048
	**day 100**	ATG-F	-1.18	(0.35)	0.0017
	**day 180**	ATG-F	-1.77	(0.58)	0.0045
	**day 365**	ATG-F	-1.71	(0.48)	0.0016

Tested variables included: use of pre-transplant ATG-F, patient’s age, donor’s age, type of donor (related or unrelated), donor/patient HLA-match (HLA-matched or HLA-mismatched), donor/patient CMV serostatus (-/-or all other combinations), CD34+ cell dose (log-transformed), postgrafting immunosuppression (cyclosporine+methotrexate, tacrolimus+methotrexate or cyclosporine/tacrolimus alone) and acute GVHD (if it occurred before the day of immune cell assessment). To minimize chance of spurious associations because of multiple comparisons, p <0.01 was considered significant. Only significant variables are shown.

### Infections

**Fig 4:**
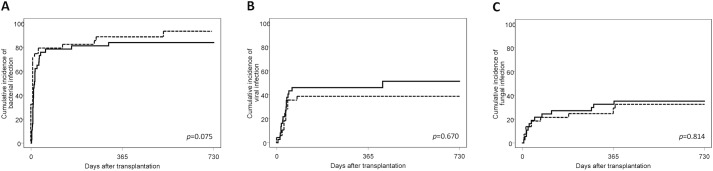
Cumulative incidence of infections after PBSCT with or without ATG-F. Cumulative incidences of bacterial (A), viral (B) and fungal (C) infections in ATG-F (solid line) and control (broken line) patients are shown.

The 2-year cumulative incidence of infection-related death was 8.1% (95% CI, 2.0–19.8%) and 10.9% (95% CI, 2.7–25.7%) in ATG-F and control patients, respectively (*p* = 0.64). No significant difference was observed in the 2-year cumulative incidences of serious bacterial, viral and fungal infections, between ATG-F and control patients (**Fig 4**). Numbers of serious infections by post-transplant time period (days 0–40, 41–180, 181–365 and 366–730) are shown in **S1 Table.** There was no difference in numbers of bacterial, viral or fungal infections between ATG-F and control patients, with the exception of the 181–365 day-period when higher numbers of bacterial infections were observed in control patients. No case of PTLD was diagnosed during the study period. Two EBV-viremia were observed after 2006. Both of them occurred in patients who received pre-transplant ATG-F, during the early post-transplant period (0–40 days after PBSCT). None of them resulted in PTLD. Three parasitic infections were observed during the study follow-up, each of them in the ATG-F group. However, because of their rarity, no conclusion might be driven for comparison between ATG-F and control patients. Overall, we did not observe higher rate of total serious infections in ATG-F in comparison with control patients. However, a confounding factor in our analysis might have been the higher incidence of acute GVHD in control patients since GVHD (and its treatment) is a known risk factor for infections.

## Discussion

Although pre-transplant infusion of ATG-F is broadly used for GVHD prevention after allogeneic PBSCT, its impact on immune reconstitution is still poorly documented. Here, we reported longitudinal monitoring of immune recovery after myeloablative PBSCT and assessed the impact of pre-transplant ATG-F. We observed that pre-transplant infusion of ATG-F had a long-lasting impact on reconstitution of the T-cell pool in the peripheral blood. However, ATG-F patients did not experience higher incidence or higher rate of serious infections after PBSCT.

In comparison with previous reports having focused on ATG-T [16, 17], we observed that pre-transplant infusion of ATG-F similarly impaired recovery of naive CD4^+^ and naive CD8^+^T cells up to at least 1 year after PBSCT. Looking precisely at memory T cells, we observed that ATG-F also selectively depleted central memory CD4^+^T cells but did not compromise effector memory CD4^+^ and CD8^+^T-cell recovery. ATG-F was even associated with compensatory higher proportion of effector memory CD8^+^ T cells, mainly because of naive cell depletion. ATG-F did neither decrease levels of Treg, B, iNKT nor NK cells. Mechanisms by which pre-transplant ATG-F selectively impairs naïve T-cell and central memory CD4^+^T-cell recovery are not well documented. It is now widely accepted that reconstitution of the T-cell pool after transplantation arises from both homeostatic peripheral expansion (HPE) of donor T cells passively transferred with the graft, and naive T-cell neo-production by the thymus (reviewed in[34, 35]). In patients given high-intensity conditioning, most circulating T cells during the first months post-transplantation are the progeny of HPE. Based on previous pharmacokinetic analyses having shown that ATG preparations persist for several weeks after administration[8, 9], it can be postulated that pre-transplant ATG-F *in vivo* modulates the composition of the graft by depleting subsets of donor immune cells early after graft infusion. Our results suggest that ATG-F selectively depletes donor naive T cells and central memory CD4^+^T cells, while it relatively spares other T cells. Consistently, differential cytotoxic effects of ATG have been reported in the setting of solid organ transplantation and demonstrated *in vitro*[36]. Whether this is due to selective effects of ATG or to cell-specific intrinsic resistance to ATG-mediated depletion is unknown. Moreover, it is also known that HPE occurs asymmetrically among T cells, with CD8^+^T cells (especially memory cells) being more prone to undergo HPE than CD4^+^T cells and naive T cells[37]. Hence, such differences in HPE propensity among T-cell subsets may have subsequently maintained, or even amplified, long-term differences in T-cell subset recovery. In addition, a third hypothesis for the relative depletion of naive T-cell compartment and compensatory expansion of effector memory T-cell compartment in ATG-F patients may be that ATG-F induces switch in cell phenotype, given that ATG-F also contains activating T-cell antibodies (i.e. anti-CD3 antibodies) that could promote a memory phenotype.

Levels of naive CD4^+^T cells in ATG-F patients were low up to day 120 after PBSCT and began to increase from day 180–365 after PBSCT. This tempo likely corresponds to new waves of naive cells originating from the thymus. Hence, we evaluated ATG-F effects on thymic-dependent T-cell neo-generation by both sjTRECs quantification and flow cytometry assays. By comparison with control patients, patients who received pre-transplant ATG-F had lower levels of circulating RTE on day 100. Similar results were reported after ATG-T-conditioned transplantation [17, 19]. It is not excluded that ATG-F might have impaired early thymopoieisis (prior to day 100). However, because thymic-dependent T-cell neo-generation after myeloablative transplantation almost takes place beyond day 100[35, 38], another plausible hypothesis is that the low levels of RTE in ATG-F patients on day 100 might have also resulted from peripheral destruction of donor RTE that had been passively transferred with the graft. Thereafter, no more difference was observed in RTE levels on day 365 and their counts significantly increased from day 100 to day 365 in ATG-F patients, suggesting that ATG-F unlikely impairs thymopoiesis at 1-year after PBSCT. To the contrary, such significant increase in RTE levels from day 100 to day 365 was not observed in control patients. Hypothesis might be lower thymic output in these patients because of higher rate of acute GVHD [39]. Therefore, ATG-F might have helped to maintain thymic functions after PBSCT by preventing GVHD-induced thymic damages.

As discussed above, we observed that use of pre-transplant ATG-F significantly modified the composition of circulating T cells up to at least 6–12 months after PBSCT, with most CD4^+^ and CD8^+^T cells in the peripheral blood of ATG-F patients being of effector memory phenotype while naive cells being almost absent. It is known that effector memory cells play major role in transfer of anti-pathogen T-cell memory from the donor to the recipient after transplantation [40]. There is also accumulating evidence that effector memory T cells poorly mediate GVHD reactions [40–42] and that donor T cells that promote GVHD mainly reside within the naive T cell compartment [43, 44]. Hence, the early shift in T-cell phenotype in favor of effector memory and disfavor of naive subsets after ATG-F might be one of the mechanisms by which use of pre-transplant ATG-F did not result in higher incidence/rate of serious infectious complications but contributed to lower incidence of acute GVHD in our cohort. In addition, it appeared in our study that ATG-F spared B and NK cells, which might also have contributed to anti-pathogen defenses. On the other hand, Treg were similarly not affected by pre-transplant ATG-F. Since these cells can mediate immune tolerance [45, 46], their persistence might have also prevented GVHD. Eventually, as discussed above, naive T cells that emerged only tardily (6–12 months) after ATG-F PBSCT were likely RTE that have been educated in the recipient’s thymus. Hence, these cells were likely tolerant to recipient’s tissues antigens and less prone to promote GVHD.

Clinical outcomes of our cohorts have obviously been influenced by improvements in clinical practice and supportive care that occurred over the study period (from 2000 to 2012). Since distribution of ATG-F and control patients was not symmetrical according to date of PBSCT, it is plausible that the period of PBSCT also contributed to lower incidences of non-relapse mortality and infection among ATG-F patients. To the contrary, it is unlikely that the period of PBSCT impacted GVHD incidence and immune recovery. Because of the retrospective nature of our study and the heterogeneity of our cohorts, no firm recommendation can be driven from this study concerning the use of ATG-F before PBSCT in the clinic. The aim of this study was to assess the imprint of pre-transplant ATG-F on the circulating immune cell pool after myeloablative PBSCT and to raise some hypotheses about the mechanistic pathways by which pre-transplant ATG-F may influence post-transplant outcomes.

In conclusion, our study suggests that pre-transplant ATG-F induces long-lasting modulation of the circulating immune cell pool after myeloablative PBSCT. Whether this may constitute one mechanism by which ATG-F prevents GVHD reactions without deeply compromising anti-pathogen defenses has to be confirmed in further studies.

## Supporting Information

S1 Fig Outcomes after PBSCT with or without ATG-F. A) Kinetics of donor T-cell chimerism in patients who received (blue box) or not (white box) pre-transplant ATG-F. Box and whisker plots display the median, 25th and 75th percentiles of the distribution (box) and whiskers extend to 5th and 95th percentiles.; B-F) Post-transplant outcomes of patients who received (solid line) or not (broken line) pre-transplant ATG-. Overall survival (B) and cumulative incidences of non-relapse mortality (NRM) (C); relapse (D); grade II-IV acute GVHD (E) and moderate/severe chronic GVHD (F).
(TIF)

S2 Fig Treg, B, NK and iNKT cell recovery after PBSCT with or without pre-transplant ATG-F. Levels of circulating Treg (A, B), B cells (C-E), NK cells (F, G) and iNKT cells (I) in the peripheral blood of ATG-F (blue box) and control (white box) patients. Circulating immune cells phenotypes were assessed for 31/34, 26/30, 28/29 and 20/25 disease-free survivors in the ATG-F cohort on days 40, 100, 180 and 365 after PBSCT, respectively; and for 6/23, 15/17, 9/15 and 7/9 disease-free survivors in the control cohort on days 40, 100, 180 and 365 after PBSCT, respectively. Box and whisker plots display the median, 25th and 75th percentiles of the distribution (box) and whiskers extend to 5th and 95th percentiles. The grey horizontal line and shaded grey area show the median and normal range (from 5th to 95th percentile) in 22 age-matched healthy controls.
(TIF)

S1 Methods Prophylaxis against infections after PBSCT. (PDF)

S2 Methods sjTRECs quantification assay. (PDF)

S1 Table Numbers of serious infectious events by post-transplant time period. (PDF)
