# Supplementary figures and images for: Impact of Pre-Transplant Anti-T Cell Globulin (ATG) on Immune Recovery after Myeloablative Allogeneic Peripheral Blood Stem Cell Transplantation
Source: PLoS One. 2015 Jun 22;10(6):e0130026. doi: 10.1371/journal.pone.0130026 (PMC4476691; doi:10.1371/journal.pone.0130026)

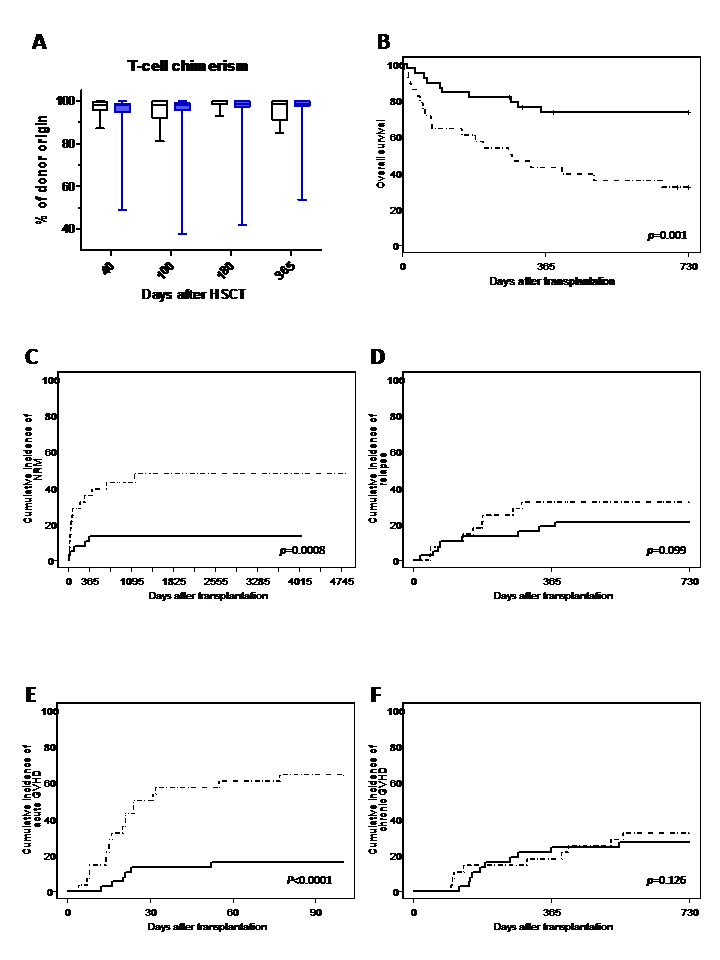

Supplement: S1 Fig — A) Kinetics of donor T-cell chimerism in patients who received (blue box) or not (white box) pre-transplant ATG-F. Box and whisker plots display the median, 25th and 75th percentiles of the distribution (box) and whiskers extend to 5th and 95th percentiles.; B-F) Post-transplant outcomes of patients who received (solid line) or not (broken line) pre-transplant ATG-. Overall survival (B) and cumulative incidences of non-relapse mortality (NRM) (C); relapse (D); grade II-IV acute GVHD (E) and moderate/severe chronic GVHD (F). (TIF) [file pone.0130026.s001.tif]

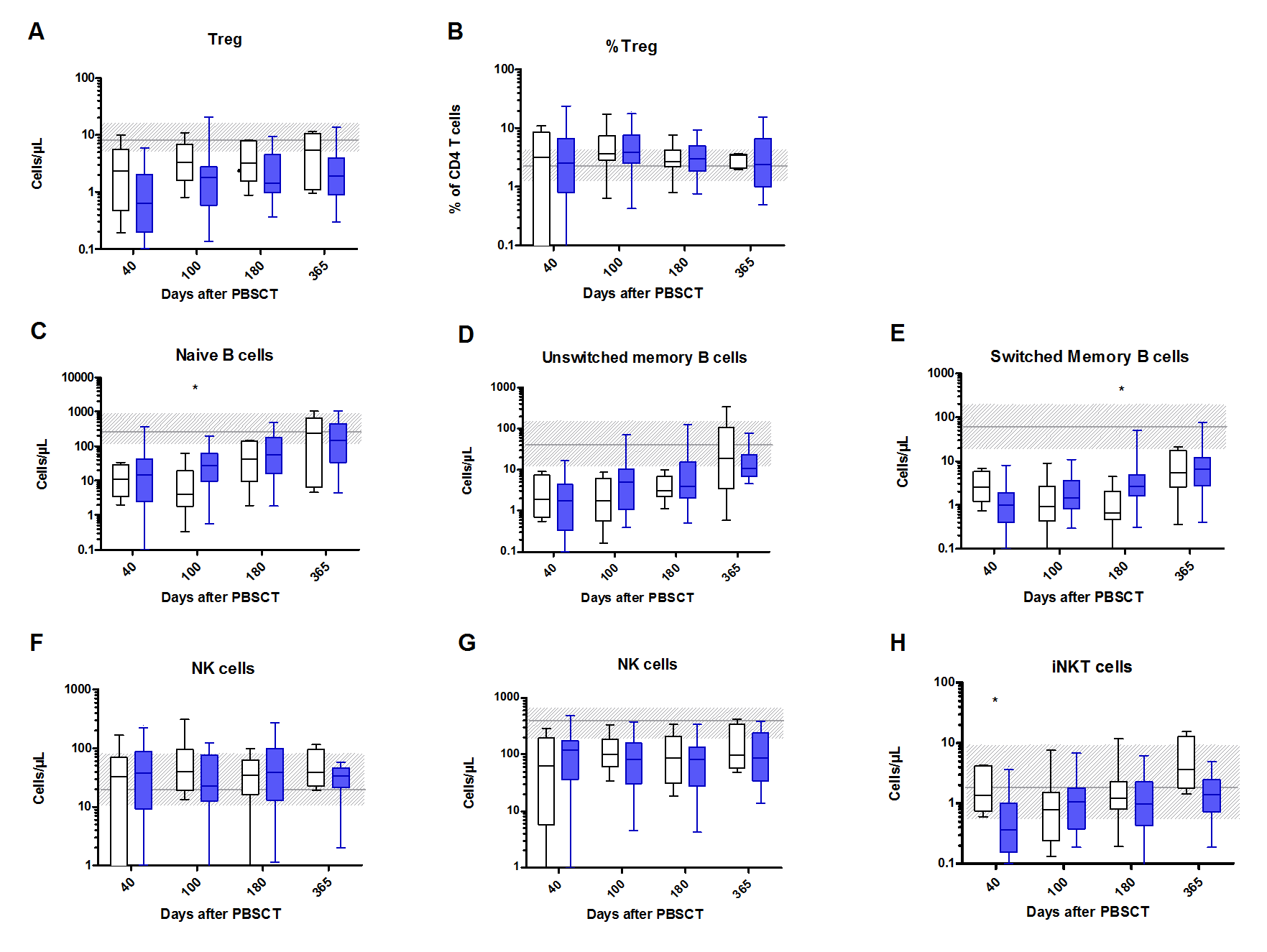

Supplement: S2 Fig — Levels of circulating Treg (A, B), B cells (C-E), NK cells (F, G) and iNKT cells (I) in the peripheral blood of ATG-F (blue box) and control (white box) patients. Circulating immune cells phenotypes were assessed for 31/34, 26/30, 28/29 and 20/25 disease-free survivors in the ATG-F cohort on days 40, 100, 180 and 365 after PBSCT, respectively; and for 6/23, 15/17, 9/15 and 7/9 disease-free survivors in the control cohort on days 40, 100, 180 and 365 after PBSCT, respectively. Box and whisker plots display the median, 25th and 75th percentiles of the distribution (box) and whiskers extend to 5th and 95th percentiles. The grey horizontal line and shaded grey area show the median and normal range (from 5th to 95th percentile) in 22 age-matched healthy controls. (TIF) [file pone.0130026.s002.tif]
