# Supplementary material for: Impact of Pre-Transplant Anti-T Cell Globulin (ATG) on Immune Recovery after Myeloablative Allogeneic Peripheral Blood Stem Cell Transplantation
Source: PLoS One. 2015 Jun 22;10(6):e0130026. doi: 10.1371/journal.pone.0130026 (PMC4476691; doi:10.1371/journal.pone.0130026)
Supplement: S1 Methods — (PDF) [file pone.0130026.s003.pdf]

S1 Methods. Prophylaxis against infections after PBSCT.

According to institutional guidelines, levofloxacine was discontinued when patients had an absolute neutrophil count above 1 x 10^9/L. Antifungal prophylaxis was stopped at day 100 if patients had an absolute $CD4^+$ T-cell count above 0.2 x 10^9/L and were off corticosteroids. Prophylaxis against Pneumocystis Jiroveci and Toxoplasmosis was withdrawn when immunosuppressive treatment was discontinued and $CD4^+$ T-cell count was $\geq$ 0.2 x 10^9/L. Acyclovir was continued for at least 6 months after cessation of immunosuppression. Posaconazole and oral amoxicillin were given in case of moderate/severe chronic GVHD.
