# Supplementary material for: Impact of Pre-Transplant Anti-T Cell Globulin (ATG) on Immune Recovery after Myeloablative Allogeneic Peripheral Blood Stem Cell Transplantation
Source: PLoS One. 2015 Jun 22;10(6):e0130026. doi: 10.1371/journal.pone.0130026 (PMC4476691; doi:10.1371/journal.pone.0130026)
Supplement: S2 Methods — (PDF) [file pone.0130026.s004.pdf]

S2 Methods. sjTRECs quantification assay.

Thawed PBMCs were lysed 30 min at 56°C in a TRIS buffer at pH 8 added with Tween-20 (0.05%), IgePal(0.05%) and Proteinase K (100 µg/ml). Multiplex PCR amplification was achieved for sjTREC together with the CD3 γ/chain, used as a housekeeping gene, with specific 3'/5' outer primers for each amplicon. Primer/probe sequences have been reported elsewhere (Poulin et al. Blood 2003, reference 26 in main article). Quantitative PCR was performed by using the Lightcycler™ technology. Quantitative PCR conditions were: 5 min initial denaturation at 95°C followed by 40 cycles of amplification (5 second at 95°C, 15 seconds at 60°C, 10 seconds at 72°C). Fluorescence emissions were assessed after the hybridization steps. Each PCR product was run for both sjTREC and CD3/γ chain in two separate Lightcycler experiments. Every sample was run at least in 3 independent experiments. Because each PBMC contains 2 CD3/γ chain copies, the number of sjTRECs per 10^5 PBMCs was calculated as followed = (sjTRECs/CD3γ) x 2 per 10^5. The results were first calculated as absolute numbers of sjTRECs per 10^5 PBMCs and sjTRECs concentration (per mL) in peripheral blood was computed using the following formula: [number of sjTRECs per 10^5 PBMCs x PBMCs/µl)]/100, where PBMCs/µl = white blood cells /µl x (%lympho + %mono). The nested character of this quantitative PCR allows high sensitivity (detection of 1 copy of sjTREC per PCR reaction).
