# Supplementary material for: Impact of Pre-Transplant Anti-T Cell Globulin (ATG) on Immune Recovery after Myeloablative Allogeneic Peripheral Blood Stem Cell Transplantation
Source: PLoS One. 2015 Jun 22;10(6):e0130026. doi: 10.1371/journal.pone.0130026 (PMC4476691; doi:10.1371/journal.pone.0130026)
Supplement: S1 Table — (PDF) [file pone.0130026.s005.pdf]

S1 Table. Numbers of serious infectious events by post-transplant time period.

	ATG	Control	
Period	Number of infections; patients with one or more infections	Number of infections; patients with one or more infections	*p*
**0-40 days**			
**Total**	**53 infections; 30/34 (88%)**	**35 infections; 21/23 (91%)**	0.99
Bacterial	30 infections; 27/34 (79%)	22 infections; 18/23 (78%)	0.67
Viral	15 infections; 13/34 (38%)	8 infections; 8/23 (35%)	0.68
Fungal	7 infections; 7/34 (21%)	5 infections; 5/23 (22%)	0.93
Parasitic	1 infection; 1/34 (3%)	0 infection; 0/23	-
**41-180 days**			
**Total**	**30 infections; 14/29 (48%)**	**14 infections; 5/13 (38%)**	0.72
Bacterial	10 infections; 8/29 (28%)	8 infections; 3/13 (23%)	0.94
Viral	17 infections; 8/29 (28%)	5 infections; 4/13 (31%)	0.92
Fungal	2 infections; 2/29 (7%)	1 infection; 1/13 (8%)	0.95
Parasitic	1 infection; 1/29 (3%)	0 infection; 0/13	-
**181-365 days**			
**Total**	**12 infections; 8/25 (32%)**	**14 infections; 7/9 (78%)**	0.025
Bacterial	4 infections; 4/25 (16%)	12 infections; 5/9 (56%)	0.015
Viral	6 infections; 3/25 (12%)	1 infection; 1/9 (11%)	0.89
Fungal	1 infection; 1/25 (4%)	1 infection; 1/9 (11%)	0.47
Parasitic	1 infection; 1/25 (4%)	0 infection; 0/9	-
**366-730 days**			
**Total**	**7 infections; 7/25 (28%)**	**3 infections; 3/7 (43%)**	0.48
Bacterial	5 infections; 5/25 (20%)	2 infections; 2/7 (29%)	0.66
Viral	2 infections; 2/25 (8%)	0 infection; 0/7	-
Fungal	0 infection; 0/25	1 infection; 1/7 (14%)	-
Parasitic	0 infection; 0/25	0 infection; 0/7	-

Serious infectious events were analyzed in patients who survived free of progression at the end of the concerned period.
